# Mitochondrial Dynamics in Brain Cells During Normal and Pathological Aging

**DOI:** 10.3390/ijms252312855

**Published:** 2024-11-29

**Authors:** Vladimir S. Sukhorukov, Tatiana I. Baranich, Anna V. Egorova, Anastasia V. Akateva, Kseniia M. Okulova, Maria S. Ryabova, Krisitina A. Skvortsova, Oscar V. Dmitriev, Natalia M. Mudzhiri, Dmitry N. Voronkov, Sergey N. Illarioshkin

**Affiliations:** Laboratory of Neuromorphology, Brain Science Institute, Research Center of Neurology, Moscow 125367, Russiaav_egorova@bk.ru (A.V.E.); nastya.akateva.05@mail.ru (A.V.A.); kseniaokul2101@gmail.com (K.M.O.); ryabovamarias@gmail.com (M.S.R.); skvortsova_ka@mail.ru (K.A.S.); dmitrievoskar87@gmail.com (O.V.D.); mudzhiri@neurology.ru (N.M.M.); voronkov@neurology.ru (D.N.V.); snillario@gmail.com (S.N.I.)

**Keywords:** mitochondrial dynamics, neurodegeneration, mitophagy, neuroprotection

## Abstract

Mitochondrial dynamics significantly play a major role in the pathogenesis of neurodegenerative diseases, such as Parkinson’s disease and Alzheimer’s disease. The dysregulation of mitochondrial biogenesis and function, characterized by impaired fission and fusion processes mediated by a number of proteins, in particular, Drp1, Mfn1, Mfn2, Opa1, and PGC-1α, contributes to neuronal vulnerability and degeneration. Insufficient mitophagy and disrupted mitochondrial transport exacerbate oxidative stress and neurotoxicity. Emerging therapeutic strategies that target mitochondrial dynamics, including various pharmacological agents, demonstrate potential for restoring mitochondrial balance and enhancing neuroprotection. This growing body of research underscores the importance of mitochondrial health in developing effective interventions for neurodegenerative conditions. This review highlights well-established links between the disruption of mitochondrial dynamics and the development of neurodegenerative processes. We also discuss different therapeutic strategies that target mitochondrial function in neurons that have been proposed as perspective neuroprotective treatments.

## 1. Introduction

It is inevitable that age-related changes will manifest in all organs and systems of the human body. Particularly significant are the processes occurring during the aging of the nervous system, accompanied by atrophic processes that may vary in their degree of severity. The aging of the brain is a programmed physiological process that is characterized by the development of cognitive disorders and other neurodegenerative manifestations. Among the pathogenic mechanisms of neural tissue damage in age-related or pathologically early neurodegeneration, mitochondrial disorders play a significant role.

Neurons are especially susceptible to mitochondrial dysfunction due to their reliance on a healthy pool of these organelles. A sufficient number of mitochondria not only maintain the activity of neurons, providing them with sufficient amounts of ATP, but also protect the cells from oxidative damage and perform a multitude of other functions vital for cell survival [1]. While the majority of these functions are typical of all cell types, in neurons, the mitochondria participate in some specific processes. For instance, they play a role in regulating synaptic plasticity, a complex phenomenon that in turn determines the efficacy of information transfer between different parts of the nervous system and beyond. On the other hand, mitochondrial damage can be highly detrimental to cell vitality, for example, through the excessive production of reactive oxygen species (ROS) [2]. Thus, the normal functioning and state of mitochondria in neurons are of great importance, as they are susceptible to change with age and pathological neurodegeneration processes [3].

### 1.1. Mitochondrial Dynamics

Mitochondria, which are general-purpose organelles found in virtually all eukaryotic cells, are currently the subject of great research interest driven by the desire for a deeper understanding of normal tissue functioning and the mechanisms of pathogenesis of various diseases, as well as finding new methods of their diagnosis and treatment. One of the reasons for this growing interest in mitochondria is the deciphering of the basic molecular mechanisms of mitochondrial homeostasis, which encompass a complex of changes united by the term “mitochondrial dynamics”. In the context of morphological studies, this complex is primarily considered in terms of quantitative alterations in these organelles, involving two principal processes: fission and fusion. The function of mitochondrial fission depends on its form: if fission occurs in the center of the organelle acting as proliferation, it is necessary to increase the number of mitochondria; conversely, if fission involves detachment of peripheral fragments, the purpose of this process is the utilization of pathological material [4]. Mitochondrial fusion is thought to facilitate the constant exchange of proteins, metabolites, and mtDNA necessary for chondriome synchronization and promote increased oxygen consumption, oxidative phosphorylation, and consequently increased ATP production [5,6]. It has been observed that fusion can result in the formation of elongated or ring-shaped mitochondria if end-to-end fusion has occurred, as well as T-shaped mitochondria in the case of lateral fusion [7].

In recent years, a large number of studies have been devoted to deciphering these mechanisms. In many respects, this interest is determined by the growing number of observations indicating the important role of changes in proteins regulating mitochondrial dynamics in the pathogenesis of many disorders, including those associated with aging and pathological neurodegeneration. Before proceeding to a review of the relevant studies, let us briefly consider what is currently known about the physiological role of some of these proteins.

The key events of the first stage of mitochondrial biogenesis are transcriptional and translational synthetic processes occurring both directly in mitochondria and in the nucleus. The main trigger of these events is the PGC-1α protein (the other members of this family, PGC-1 and PGC-1β, also play an important role in mitochondrial biogenesis). PGC-1α triggers the activation of a number of nuclear transcription factors, such as Nrf-1 and Nrf-2 (Nuclear respiratory factors 1 and 2), Err-α (Estrogen-related receptor-α), and Tfam (Mitochondrial transcription factor A), a direct stimulator of mtDNA transcription and replication [8,9]. Being one of the main regulators of mitochondrial biogenesis, PGC is actively expressed in tissues with high energy demands. Maintaining an appropriate energy level in cerebral neurons is essential for the normal functioning of the brain. Chronic cerebral hypoperfusion in transgenic mice overexpressing PGC-1α is characterized by a decrease in PGC-1α levels in the hippocampus accompanied by cognitive impairment. The activation of PGC-1α after chronic hypoperfusion in mice leads to an improvement in their learning abilities, whereas PGC-1α knockdown exacerbates the impairments. The neuroprotective effect of PGC is likely attributed to its modulation of neuronal metabolic activity and reduction of ROS production, as well as suppression of inflammation through its effect on the neuroglial cells [10].

Dynamin-related protein 1 (Drp1) is a cytoplasmic GTPase that initiates mitochondrial fission after its recruitment to the outer mitochondrial membrane, and at least one of the conditions for fission initiation is establishing contact between the mitochondrial membrane and membranes of the endoplasmic reticulum [11,12]. It is hypothesized that the selection of the fission mechanism depends on the interaction of Drp1 with the outer mitochondrial membrane proteins Mff or Fis1. The mitochondrial outer membrane protein Mff (mitochondrial fission factor) is responsible for initiating the proliferation process following preliminary compression of the mitochondrion by actin filaments (“pre-constriction”). On the contrary, the fragmentation process is initiated by the interaction of Drp1 with the mitochondrial outer membrane protein Fis1 (Fission 1) following prior contact with the lysosome [4,13,14,15]. In turn, Drp1 wraps around the organelle and ruptures the latter at the expense of GTP energy.

In the context of this review, it is important to highlight some distinctive features of mitochondrial dynamics in neurons. Thus, Lewis et al. demonstrated the role of Mff-dependent mitochondrial fission in maintaining the reduced size of these organelles, which is necessary for their correct neuronal transport, for the regulation of synaptic transmission, and even for efficient axonal branching. Mff ex-utero knockdown has been shown to result in a fourfold increase in the length of mitochondria transported from the soma of cortical neurons to the axon and along the axon. Interestingly, in normal cortical neurons, mitochondrial morphology varies depending on their localization: in particular, axonal mitochondria are shorter in length than dendritic ones. These findings suggest that Mff-dependent mitochondrial fission is involved in the two-step maintenance of axonal mitochondrial length constancy: at the axon entrance from the soma and along the axon. The knockdown of Mff by electroporation in utero disrupted terminal axon branching, while axon formation and growth did not differ from the control. In addition, the authors demonstrated that reduced Mff expression affects calcium dynamics in presynaptic endings. Mff knockdown resulted in increased mitochondrial calcium buffering, decreased levels of calcium in presynaptic terminals, impaired synaptic vesicle exocytosis, and, as a result, impaired synaptic transmission [16]. It has been previously suggested that spontaneous neurotransmitter release supports axonal branching [17,18,19]. Thus, impaired axon branching in Mff knockdown can be explained by reduced synaptic transmission activity. Importantly, reduced Mff expression had no effect on ATP production, mitochondrial transport along the axon, their membrane and redox potential, and the transport of these organelles from axons to presynaptic terminals [16].

Mitochondrial outer membrane protein mitofusins (Mfn1 and Mfn2) are involved in the process of GTP-dependent mitochondrial fusion [20]. Specifically, Mfn1 regulates the fusion of the outer membranes of two mitochondria, while Mfn2 is responsible for the formation of MAM (mitochondria-associated membrane) complexes with the endoplasmic reticulum [12,21]. Cellular redox activity is believed to play a triggering role in the initiation of mitochondrial fusion, but the key mechanism of this process is associated with mitofusins, as the oxidation of cysteine residues in their cytoplasmic segments results in their oligomerization, leading to the fusion of outer mitochondrial membranes [22,23]. Interestingly, Mfn1 has greater “binding capacity” as well as increased GTPase activity compared to Mfn2 [24], with Mfn1 and Mfn2 expression being tissue-specific [25]. Thus, a higher expression of Mfn2 has been observed in the brain compared to Mfn1 [26]. In addition, it has been shown that the knockout of Mfn2 in mice made after placentation leads to disruption of cerebellar development and early death, which does not occur in Mfn1 knockout [27]. Some researchers attribute such results precisely to the predominance of Mfn2 expression in brain tissue [28].

Opa1 (Optic atrophy-1) is a cytoplasmic protein that plays a regulatory role in the fusion of mitochondrial inner membranes. Upon fusion initiation, Opa1 moves into the mitochondrial intermembrane space, where the enzyme MPP (Mitochondrial processing peptidase) catalyzes its conversion into the so-called “large form” (L-OPA1) anchored in the inner mitochondrial membrane [15,24]. Subsequently, the enzymes OMA1 (Overlapping proteolytic activity with m-AAA protease 1) and YME1L (Yeast mitochondrial escape protein 1-like) convert the “large” form of Opa1 into a “small” form (S-OPA), which, unlike the former, moves freely in the intermembrane space. The fusion of inner mitochondrial membranes is initiated by L-OPA1 and cardiolipin, whereas S-OPA is required for the efficient completion of the fusion process. Apart from having both forms of Opa1 being necessary to trigger the fusion, the importance of maintaining the balance of S-OPA and L-OPA1 concentrations is also worth noting [29]. The disruption of this balance, in particular, the excessive cleavage of L-OPA1, leads to the failure of the fusion process and uncontrolled mitochondrial fragmentation [30].

### 1.2. Mitochondrial Fission in Aging

The study conducted by Li Y. [31] demonstrated that the aging process in the hippocampus and cerebral cortex of mice is accompanied by an increase in the amount of fission protein Fis1, while the concentration of Drp1 is significantly increased. Obviously, such an increase in the level of fission proteins indicates the activation of mitochondrial fragmentation, especially if we take into account that Fis1 enhances Drp1 aggregation in these organelles [32]. We can speculate that activated fragmentation is necessary for the elimination of the defective fragments of these organelles that accumulate during aging. However, it has been suggested that the rise in the number of mitochondrial fission proteins may disrupt the mitochondrial membrane potential and inhibit the respiratory chain, which leads to slower cell growth and accelerates neuronal aging [33]. Especially worth mentioning are the critical consequences of these processes for synapse preservation, where the amount of the activated (phosphorylated) form of Drp1 increases with age [34], suggesting a potential role for alterations in synaptic bioenergetics in the development of age-related neurotransmission disorders.

Mishra et al. analyzed the expression of mitochondrial dynamics proteins in the hippocampus of young, adult, and old mice at 10, 30, and 80 months of age, respectively. The immunoblotting of hippocampal homogenates demonstrated a decrease in the level of phospho-Drp1 at S616 in adult mice and then its increase in old animals compared to the control group of young mice [2]. According to Zhou et al., the phosphorylation of Drp1 at S616 activates Drp1, promotes mitochondrial translocation of the protein, resulting in mitochondrial fragmentation, and is possibly involved in neuronal death [35], suggesting that the increased level of phospho-Drp1 correlates with the mitochondrial morphological changes in the hippocampus and cognitive decline observed with aging.

Another study revealed a mechanism of posttranscriptional inhibition of Mff synthesis through binding of the ribonucleoprotein PUM2 (Pumilio homolog 2). PUM2 expression has been shown to increase during aging in the mouse neocortex. The nucleotide sequence of *Mff* mRNA showed an exact match with the known *PBE* (Pumilio-binding-element) sequence, and decreased PUM2 expression in mouse myoblasts and HeLa cells led to an increase in basal and maximal levels of cellular respiration, while Mff expression was increased at the protein but not the mRNA level. The latter might indicate the post-transcriptional regulation of Mff expression. Meanwhile, previously, an inverse correlation between PUM2 expression in the neocortex and the lifespan of mice in BXD and LXS reference populations was reported [36]. Thus, the PUM2-dependent inhibition of Mff may be responsible for the mitochondrial dysfunction that develops during aging. Moreover, in light of the results by Lewis et al. [16], cited in the previous section, this is primarily due to impaired transport of relatively large mitochondria along axons, which subsequently results in insufficient energy exchange in synapses.

### 1.3. Mitochondrial Fusion in Aging

It has long been demonstrated that mitochondrial fusion impairment resulting from decreased mitofusin expression is associated with slow cell growth, impaired cellular respiration, heterogeneous mitochondrial population, and decreased membrane potential [37]. These effects are characteristic of brain aging and, in particular, may play a significant role by damaging the population of neuronal precursors. Some authors proposed a link between the aging process, as well as the pathogenesis of neurodegenerative disorders, with the loss of stem cells [38,39], but the causes of this phenomenon remain largely unexplored. Khacho M. et al. investigated the significance of Mfn1 and Mfn2 in the differentiation and survival of neural progenitors in in vitro, in vivo, and in utero models. To evaluate the effect of mitochondrial dynamic disruptions on stem cell self-renewal potential under in vitro conditions, the authors used the positive correlation of this parameter with the number of neurospheres formed by Sox2+ non-committed stem cells. They showed that the knockout of *Mfn1/2* as well as the knockdown of *Opa1* led to a decrease in the number of neurospheres, but these changes were more pronounced in the case of *Mfn1/2*. The opposite dynamics were observed for *Drp1* knockout. The knockout of *Mfn1/2* in early terminal brain neurons performed in vivo before the onset of neurogenesis resulted in increased mitochondrial fragmentation, increased Sox2+ stem cell division rate, and a change in the division plane from vertical to predominantly horizontal [40]. In earlier studies, such a change in the division plane was shown to correlate with a decrease in the self-renewal potential of stem cells [41]. It is important to note that *Mfn1/2* knockout was performed at 9.5–10.5 days of embryogenesis, while the changes were observed as early as 12.5 days, which excludes the influence of the effects of prolonged mitochondrial dysfunction and secondary energy deficiency on the obtained results. The authors demonstrated that the in-utero *Opa1* knockdown in the dorsal forebrain decreases the self-sustaining ability of neuronal progenitors due to a decrease in their potential for multidirectional differentiation. These findings prove the direct effect of maintaining balanced mitochondrial dynamics on stem cell differentiation. This hypothesis is also supported by the similarity of the bioenergetic profile of mitochondria of Sox2+ stem cells with that of *Mfn1/2* knockout and committed Tbr2+ cells. A lack of *Mfn1/2* expression resulted in a reduced pool of non-committed Sox2+ cells and young DCX+ neurons in the dentate gyrus of the hippocampus in adult mice. Moreover, the use of the Morris water maze showed impaired spatial and reversal learning in these animals. The authors argue that these results demonstrate the high sensitivity of neural stem cells to changes in mitochondrial dynamics and, consequently, indicate a link between impaired mitochondrial dynamics and the observed decrease in the pool of neural progenitors as well as cognitive impairment in normal aging and neurodegenerative disorders. It is suggested that the effect of mitochondrial dynamics on neural stem cell differentiation is ROS-mediated, implementing a regulatory cascade involving NRF (nuclear respiratory factor) that ultimately alters transcriptional processes in the nucleus. Thus, the consequence of Mfn2 and *Opa1* knockout was an increase in the mitochondrial and cytoplasmic ROS levels. The opposite dynamic was observed in *Drp1* knockout. The activation of genetic programs of neuronal progenitor differentiation was demonstrated by RNA sequencing both on models of increased ROS production and on stem cells with losses of Mfn1/2 and Opa1 [40].

As mentioned above, Opa1 is a mitochondrial inner membrane fusion protein and plays a key role in neuronal adaptation to normal and pathological aging. In their study, Bevan et al. [42] demonstrated that *Opa1* haploinsufficient mice are prone to premature aging and cognitive decline, including memory and learning deficits. The CA1 region of the hippocampus of these old mice shows a decrease in synaptic density and loss of spiking. These changes may be explained by energy dysregulation in neurons due to the loss of the Opa1 fusion protein.

As previously stated, after transport from the cytoplasm to the mitochondrial intermembrane space, Opa1 is converted into its long (L-OPA1) and short (S-OPA1) forms, which play a key role in mitochondrial membrane fusion and maintenance of cristae structure. In a mouse model of neurodegeneration, Anne Korwitz et al. [42] demonstrated that animals with a genetic deletion of the OMA1 enzyme, which cleaves L-OPA1, in the dentate gyrus of the hippocampus exhibit more delayed processes of neurodegeneration and neuroinflammation, late neuronal death, and increased lifespan compared to the control mice models of neurodegeneration without *OMA1* deletion. At the ultrastructural level, long fused mitochondria were observed, but their cristae structure was not preserved. These results suggest that the long form of L-OPA1 may play one of the key roles in cell survival and adaptation to stress, while the stress-induced activation of OMA1 promotes mitochondrial fragmentation and neuronal death.

Another aspect of mitochondrial dynamics was investigated by Anand et al. [43] in the context of cellular stress associated with the endoplasmic reticulum (so-called “ER-stress” induced by the accumulation of proteins that have undergone improper folding in the ER). They showed the involvement of vitamin B7 in the adaptation of astrocytes to ER-stress through altered mitochondrial dynamics. The metabolic coupling of ER, mitochondria, and biotin is evidenced by the ER-stress-induced decrease in the level of biotinylated mitochondrial carboxylases, which is subsequently restored following the addition of biotin to the cell culture. This phenomenon can be attributed to the heightened demand of neurons for vitamin B7, which is vital for adaptive response. Moreover, it has been shown that changes in mitochondrial dynamics as a result of ER-stress are similar to the corresponding changes during normal aging. Thus, the immunoblotting of large hemisphere cortex lysates from a group of old rats at 24 months of age showed decreased levels of both Mfn2 and Drp1 relative to young rats at 4 months of age. The same changes in mitochondrial dynamics were observed in the study of a primary culture of astrocytes, subjected to tunicamycin-induced ER-stress. Following the treatment of the cells with biotin, an increase in Mfn2 was observed, while no change was noted in Drp1. Confocal microscopy showed the association of synthesized Mfn2 with mitochondria, confirming the recruitment of Mfn2 to mitochondria rather than just an increase in its expression. The authors argue that their results confirm the previously suggested key role of Mfn2 in the creation of contacts between mitochondria and ER (the so-called MERCs—mitochondria-endoplasmatic reticulum contacts) through the formation of MAM complexes [12,21]. MERCs are thought to regulate mitochondrial homeostasis [44], and this regulation, in particular Mfn2 levels, in turn contributes to altering the number of MERC contacts according to the cell state. Indeed, the observed decrease in Mfn2 levels during ER-stress in astrocytes may contribute to a reduction in the number of MERCs to prevent mitochondrial dysfunction, while the increase in Mfn2 levels upon treatment of cells with B7 could be due to the neutralization of the effects of ER-stress and the subsequent restoration of MERC contacts [43]. Another recent study showed that the tamoxifen-induced reduction in Mfn2 expression in astrocytes resulted in a significant decrease in the number of MERC contacts, reduced mitochondrial accumulation in perivascular processes, and local disruption of Ca^2+^ dynamics and vascular remodeling during cortical damage. At the same time, mitochondrial morphology in the perivascular processes of astrocytes had no pronounced differences compared to the control [45].

In the previous section, we presented the results of Mishra et al., who found that the level of phosphorylated Drp1 decreased with age in mice. However, the results of the immunoblotting of hippocampal homogenates for Mfn2 expression showed the opposite dynamics compared to Drp1 [2].

It can be assumed that an essential factor of longevity is the maintenance of the balance between the processes of mitochondrial fission and fusion. It has recently been shown that the simultaneous overexpression of proteins regulating both mitochondrial fission and fusion despite (or perhaps due to) an increased level of mitochondrial fragmentation in the nematode C. elegans was positively correlated with stress tolerance and longevity [46]. These data emphasize the relevance of developing therapeutic drugs that would maintain the balance of mitochondrial dynamics in order to find ways to actively increase longevity, which will be discussed further.

### 1.4. Mitochondrial Dynamics in Alzheimer’s Disease

**Figure 1:**
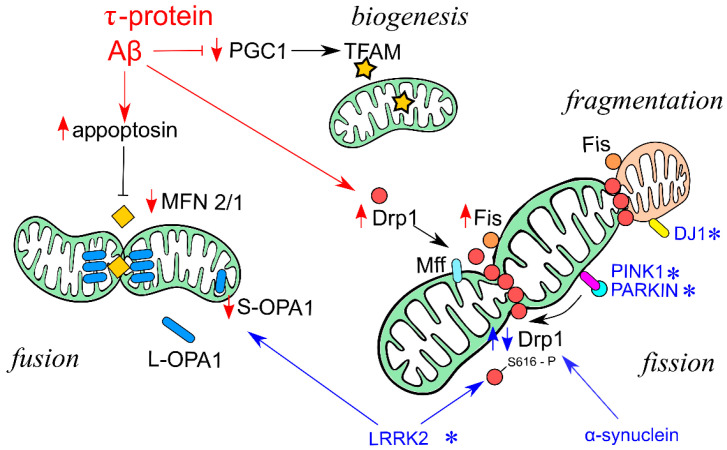
Regulatory factors of mitochondrial dynamics, associated with Alzheimer’s disease (AD) and Parkinson’s disease (PD). In AD (red arrows), beta-amyloid (Aβ) aggregation decreases PGC-1α level and thus, biogenesis of mitochondria. Aβ aggregation leads to inhibited fusion of mitochondria is inhibited through interaction of appoptosin with Mfn1/2. On the contrary, Aβ aggregation causes increased mitochondrial fission and fragmentation by elevating Drp1 and Fis1 levels. PD (blue arrows, blue asterisks) is associated with accumulation of α-synuclein and genetic forms of PD are often linked to disruptions in PINK1/PARKIN signaling pathway. PINK1 overexpression inhibits Drp1-mediated fission, while PINK1 knockdown increases mitochondrial fragmentation. Additionally, LRRK2, a genetic factor of the dominant form of PD, phosphorylates Drp1, which promotes its translocation to mitochondria and fission. LRRK2 also leads to decreased level of fusion-associated protein Opa1. Another factor of familial PD, DJ1, causes increased fragmentation of mitochondria.

The risk of developing a large number of neurodegenerative diseases, such as Alzheimer’s disease (AD) and Parkinson’s disease (PD), increases with age [47]. The disruption of mitochondrial dynamics is one of the key factors in pathological forms of neurodegeneration. The next two sections of this review are schematically summarized in Figure 1 and present our knowledge regarding the impact of mitochondrial dynamics regulation and the development of AD and PD. Changes in mitochondrial dynamics are characterized by progressive neuronal death, especially in the cerebral cortex and hippocampus. One of the first pieces of evidence to support this was the observation that a decrease in the level of PGC-1α, a key activator of mitochondrial biogenesis, correlates with neuronal death and cognitive decline in AD patients [48].

Appoptosin is a pro-apoptotic transport protein of the inner mitochondrial membrane, which has been hypothesized to play an important role in beta-amyloid-dependent neurodegeneration in AD. Treating neurons with beta-amyloid leads to an increase in appoptosin, and its role in increasing mitochondrial fragmentation has been identified [49]. Despite the involvement of appoptosin in ROS production and the initiation of apoptosis, Zhang et al. demonstrated the ROS- and caspase-independent alteration of mitochondrial dynamics mediated by this protein under in vitro conditions in HeLa cells. Moreover, the authors showed the involvement of appoptosin in the regulation of mitochondrial fusion through direct interaction with the fusion proteins Mfn1 and Mfn2 but not with Drp1 and Opa1. Appoptosin has been shown to inhibit mitochondrial fusion by preventing the formation of heterodimers of Mfn1 and Mfn2, but not their homodimers [50]. In addition to regulation of mitochondrial fusion, Mfn2 is thought to be involved in the implementation of various signaling cascades, and there have been reports indicating that Mfn2 may function as a pro-apototic and antiproliferative protein [51,52,53,54]. The involvement of Mfn2 in appoptosin-induced apoptosis is evident, as the co-expression of Mfn2 with appoptosin showed a more pronounced effect of apoptosis initiation compared to appoptosin overexpression alone, indicating a possible pro-apoptotic function of Mfn2 [50].

Later, Manczak et al.’s study demonstrated the role of beta-amyloid accumulation in the dysregulation of mitochondrial dynamics and the reduction in mitochondrial biogenesis. Thus, immunoblots of hippocampal tissue lysates from APP transgenic mice exhibited elevated levels of Drp1 and decreased levels of Mfn1, Mfn2, and Opa1. According to the authors, increased mitochondrial fragmentation impairs overall mitochondrial biogenesis. This assumption was confirmed by the observed decline in the level of PGC-1α marker [55]. The same group of authors reports a decrease in the levels of Mfn1/2 and Opa1 and an increase in the level of Drp1 in the hippocampus of 12-month-old P301L mutant mice, expressing a pathological form of tau protein compared to the controls. Furthermore, electron microscopy revealed the corresponding ultrastructural changes: an increase in the number of mitochondria and a decrease in their length [56].

Subsequent research demonstrated that impaired mitochondrial dynamics is not only a key aspect of AD pathogenesis but also an early manifestation of this neurodegenerative disorder. Misrani et al. proved that mitochondrial dysfunction in APP/PS1 transgenic mice manifests itself already at the age of 4–5 months in the form of a significant increase in the amount of Drp1 fission protein and a decrease in the level of fusion proteins Mfn1, Mfn2, Opa1 in the medial prefrontal cortex. On the other hand, in the hippocampus Drp1 levels decrease, while the levels of Mfn1, Mfn2, and Opa1 remain the same. With the aging of mice, further progression of mitochondrial dynamics impairment is observed. Thus, Western blot analysis shows an increase in fission proteins and a decrease in fusion proteins in both the medial prefrontal cortex and hippocampus. All these results demonstrate the high potential of using therapeutic approaches aimed at restoring mitochondrial dynamics in the preamyloid phase of AD [57].

Nevertheless, despite the mounting evidence confirming the role of mitochondrial dynamics in disease pathogenesis, some aspects remain inconclusive upon detailed examination. For example, Simonovitch et al. investigated the role of mitochondrial dynamics in the mechanism of pathological action of APOE4, the most important genetic risk factor for the development of Alzheimer’s disease [58]. A model of AD development was created in 4-month-old transgenic mice, which expressed human APOE3 or APOE4 isoforms. Hippocampal neurons were immunohistochemically stained to study the distribution of Mfn1 and Drp1. Increased levels of Mfn1 were observed in the hilus of the dentate gyrus as well as in the CA1 and CA3 regions of the hippocampus of APOE4 mice compared to APOE3-expressing mice, who carry a much lower risk of pathologic neurodegeneration. On the contrary, levels of Drp1 in the CA1 and CA3 regions in APOE4 mice were lower than in APOE3 mice. No significant changes in the Drp1 level were detected in the hilus of the dentate gyrus. Increased Mfn1 and decreased Drp1 levels in APOE4 mice were also demonstrated by the immunoblotting of hippocampal homogenates. To resolve the contradiction with the accumulated data presented earlier in our review, the authors attribute the observed tendency to shift the balance of mitochondrial dynamics toward fusion in young animals to a compensatory mechanism in response to cellular stress and synaptic pathology induced by APOE4 expression and suggest that the decrease in mitochondrial fusion intensity observed in elderly patients with diagnosed AD and model animals is due to an inability to compensate for neurodegenerative changes [55,58,59]. Interestingly, increased levels of Mfn1/2 and Opa1 and, consequently, a shift in the balance of mitochondrial dynamics toward fusion were also found in vitro in primary rat hippocampal neurons overexpressing wild-type tau protein. In this study, *Mfn1/2* knockdown restored normal mitochondrial and neuronal function after the disruption of mitochondrial dynamics, in contrast to Opa1 knockdown [60]. Moreover, Xu et al. demonstrated in vivo increased levels of Mfn2 in hippocampal neurons of 3- and 6-month-old APP mice modeling the development of Alzheimer’s disease. However, 3-month-old mice showed increased levels of Opa1 and Drp1, while levels of these proteins in 6-month-old mice were decreased compared to the control [61]. These findings support the aforementioned hypothesis that in cases with a hereditary risk of AD at early stages of development, the shift in the balance of mitochondrial dynamics towards fusion may serve a compensatory function.

Returning to the discussion of shifting the balance of mitochondrial dynamics in AD at more advanced stages of the disease, we note a few more studies. Djordjevic et al. [62] investigated the expression of Drp1 and Mfn2 proteins in hippocampal and cortical neurons in the AD model of 3xTg transgenic mice and showed a decrease in the level of Mfn2 protein accompanied by an increase in the level of Drp1 in the cortex of female and male 6-month-old 3xTg mice compared to the control. The authors propose that such a shift of mitochondrial dynamics towards fission may be one of the main mechanisms for the development of neurodegenerative changes in Alzheimer’s disease. However, the results obtained varied significantly depending on the age, sex, and region of the brain studied: for example, males of both groups had higher levels of Drp1 and lower levels of Mfn2 in the cortex compared to females. The obtained data show the necessity of taking into account age and sex characteristics when studying methods of diagnostics and treatment of AD.

The reduction in Mfn1/2 levels in vitro in AD modeling was also recently demonstrated by Reddy et al., who investigated the effect of beta-amyloid on mitochondrial dynamics using the HT22 cell line of hippocampal neurons. The neurons were transfected with DNA complementary to mutant APP protein (mAPP) [59]. A significant decrease in PGC-1α protein levels was shown in mAPP-HT22 cells compared to the control, an indication of reduced mitochondrial biogenesis. In addition, there was an increase in the levels of the fission proteins Drp1 and Fis1 and a significant decrease in the levels of the fusion proteins Mfn1, Mfn2, and Opa1 in transfected neurons compared to the control. The authors attributed the observed shift in mitochondrial dynamics toward fission to a physical interaction between Drp1 and beta-amyloid, leading to increased levels of Drp1 GTPase activity and ultimately to excessive mitochondrial fragmentation [59].

The above is confirmed by the results of a recent study by Wang et al., where PGC-1α expression was induced in the neurons of the lateral parietal association cortex of APP/PS1 transgenic mice by transfection with an AAV vector [63]. It was found that spatial long-term, working, and sensorimotor memory were improved under the influence of PGC-1α. The increased expression of PGC-1α in the lateral parietal association cortex prevented mitochondrial swelling and destruction in neurons, probably through correcting the rebalancing in mitochondrial dynamics. Thus, PGC-1α activation was shown to increase the levels of Opa1, Mfn1, and Mfn2 and decrease the expression of Drp1 and Fis1 [63].

A number of other studies have also demonstrated a link between the Drp1 protein and the progression of Alzheimer’s disease [34,64]: the interaction between Drp1 and the accumulation of AD toxic proteins Aβ and tau enhances the activity of Drp1, which leads to an excessive predominance of the mitochondrial fission [65,66]. These findings are corroborated by the study of the relationship between the accumulation of pathological forms of tau protein and the disruption of mitochondrial dynamics. It was demonstrated that the expression of the form of tau protein cleaved at 421D resulted in increased mitochondrial fragmentation and a reduction in Opa1 expression. The authors suggest that the disruption of mitochondrial dynamics is caused by the tau protein’s effect on mitochondrial permeability transition pores, which causes these pores to open and leads to mitochondrial failure and neurodegenerative processes in AD [67]. The publication by Kandimalla et al. demonstrates that a partial reduction in Drp1 decreases the levels of phosphorylated tau in AD and also reduces mitochondrial dysfunction, having a neuroprotective effect and enhancing synaptic activity [56]. In addition, the administration of the mitochondrial fission inhibitor Mdivi-1 in experimental AD models resulted in decreased mitochondrial fragmentation and increased mitochondrial length and improved the functioning of the organelles themselves and synapses in hippocampal neurons [64,68]. However, another study describes the formation of elongated giant mitochondria (“mitochondria-on-a-string”, MOAS phenotype), the number of which increased as AD progressed in hippocampal neurons [69]. Perhaps these organelles occur at the final stages of mitochondrial fission in order to protect against the process of mitophagy and preserve the residual function of mitochondria.

In another recent study of mitochondrial dynamics markers, the authors observed that changes in the level of fission and fusion proteins in the hippocampus occurred with delays or did not occur at all [70]. They showed decreased levels of PGC-1α, Opa1, and Mfn2 proteins in the cerebral cortex in a group of transgenic mice with AD, while no changes were detected in the hippocampus. A tendency of having low levels of Mfn1 in both the cortex and hippocampus in APP/SP1 mice was also determined, but in the hippocampus, these changes were observed in older animals. In the cortex, the decrease in Mfn2 levels was detected later than the decrease in Mfn1 levels. In contrast, the level of Drp1, a mitochondrial fission protein, increased in the cortex, while there were no changes in the hippocampus. The correlation of changes in mitochondrial dynamics with beta-amyloid accumulation was confirmed in neuronal cell cultures after they had been treated with Aβ42 for 24 h. The authors believe that later changes in the level of fission and fusion proteins or their absence can be explained by a lower level of Aβ in hippocampal neurons compared to cortex in model animals [70].

Another protein with reportedly altered activity in Alzheimer’s disease is the cytosolic CRPM2 (Collapsin response mediator protein 2) signaling molecule, which is involved in axon guidance and neurite outgrowth. The activation of CRPM2 is achieved through phosphorylation, and it has been demonstrated that the CRPM2 expression and phosphorylation correlate with the changes in mitochondrial morphology, as CRPM2 is capable of binding to Drp1 and attaching to the mitochondrial membrane [71]

The morphologic alterations of mitochondria in AD are confirmed by Wang et al., who demonstrated pathologic changes of mitochondria in pre- and postsynaptic terminals, as well as in the soma of cortical neurons [72]. The electron microscopy of cortical biopsy specimens obtained from patients with diagnosed AD revealed a significant decrease in the ratio of mitochondrial length and width, as well as in the total number of mitochondria exclusively within the soma of neurons, which, according to the authors, indicates a decrease in mitochondrial biogenesis in this compartment. In the study of mitochondrial size, a significant increase in this index was found in the postsynaptic endings of dendrites and soma. Moreover, all these changes correlated with the observed reduction in the density of synaptic vesicles in presynaptic terminals, a reduction in the number of vesicles in the reserve pool, as well as an increase in their diameter and the electron density of their contents, which suggests a direct involvement of mitochondrial dysfunction in the impairment of synaptic transmission observed in AD [72].

When assessing the characteristics of mitochondrial dynamics in Alzheimer’s disease, it is essential to take into account the zonal distribution of marker proteins. In our recent research, we obtained data on the immunohistochemical distribution of the markers PGC-1α, Mfn2, and Drp1 in the hippocampal regions of CA1, CA2, CA3, and CA4, as well as in the dentate gyrus [73]. In the CA1 region of the hippocampus on day 38 after beta-amyloid injection, a significant decrease in the mitochondrial biogenesis marker PGC-1α was observed, which may indicate a decrease in the intensity of mitochondrial biogenesis. Conversely, no significant changes were detected in the other zones. An increase in Drp1 and Mfn2 proteins was also shown in CA1, CA2, and dentate fascia regions, indicating an increase in the intensity of both mitochondrial fission and fusion in these zones. At the same time, there was an increase in Mfn2 but not Drp1 in the CA4 region, indicating a shift in mitochondrial dynamics toward fusion. However, the most pronounced changes in mitochondrial dynamics were observed in the CA3 region, where there was an increase in the amount of the fission marker Drp1 and a decrease in the fusion marker Mfn2 [73].

### 1.5. Mitochondrial Dynamics in Parkinson’s Disease

Parkinson’s disease (PD) is a multisystem neurodegenerative disorder characterized by the death of dopaminergic and adrenergic neurons in the central and peripheral nervous system and the accumulation of neurotoxic aggregated forms of α-synuclein. Despite the considerable advancement that has been made in the field of PD research, its etiology and development mechanisms remain largely unclear. It has been established that the leading pathogenetic link in both idiopathic and genetic forms of Parkinson’s disease is mitochondrial dysfunction and oxidative stress [74].

Dopamine neurons of the substantia nigra pars compacta, which are most susceptible to degenerative changes, are characterized by a low basal mitochondrial content and are projection cells with long unmyelinated axons that form a large number of synaptic contacts. The extensive synaptic field is very energetically demanding, so the vulnerability of these cells to pathological processes is explained, among other factors, by their high energy requirements against the background of low mitochondrial supply [75,76].

The process of mitophagy plays a pivotal role in the pathogenesis of neurodegenerative diseases. The insufficient utilization of damaged mitochondria or the excessive degradation of functionally active organelles leads to the disruption of cellular homeostasis, damage, and death of neurons. Mitophagy is preceded by changes in mitochondrial morphology. Thus, the mitochondrial fission mediated by Drp1 and Fis1 proteins provides its peripheral fragmentation and separates the damaged parts of the organelle for subsequent utilization in lysosomes [4].

Mutations in the genes encoding the key mitophagy proteins PINK1 (PTEN-induced kinase 1) and PARKIN (Parkinson juvenile disease protein 2) are the most common causes of autosomal recessive early-onset Parkinson’s disease. The PINK1/PARKIN signaling pathway is currently the most extensively researched with regard to its influence on mitochondrial dynamics. PINK1 regulates the latter through modifying fission proteins, as *PINK1* overexpression was found to inhibit Drp1 translation and reduce its translocation from the cytosol to the mitochondrial surface, causing the formation of elongated mitochondrial chains, whereas *PINK1* knockdown increases their fragmentation [77]. The ubiquitination of Drp1 associated with PINK1 leads to its proteasome-dependent degradation and subsequent inactivation, thus reducing the intensity of mitochondrial fission [78].

It is likely that in addition to the activation of mitochondrial biogenesis, PGC-1α also plays a neuroprotective role by reducing mitochondrial degeneration and enhancing the antioxidant protection of neurons. This allows us to consider it as a potential target in neurodegenerative diseases [9]. The above is consistent with the data collected in a mouse model of PD, which show that PGC-1α overexpression in dopaminergic neurons protects against MPTP neurotoxin-induced cell degeneration due to the increased expression of the mitochondrial antioxidants Superoxide dismutase-2 (SOD2) and Thioredoxin-2 (Trx2) [79].

It is imperative to consider mitochondrial dynamics in conjunction with the motility of these organelles, which is of particular importance in such huge cells as neurons. The PINK1/PARKIN pathway is involved in both intracellular and intercellular mitochondrial trafficking. In the event of mitochondrial damage and the subsequent loss of their membrane potential, PINK1 phosphorylates the Miro protein, causing its proteasome-dependent degradation, which leads to the suppression of mitochondrial motility [80]. Miro1, a GTPase with a high affinity for mitochondria that provides their intracellular movement and subcellular distribution, is also involved in the functioning of motor systems of intercellular mitochondrial transport [81]. This fact is especially interesting in view of the recent data on the possibility of mitochondrial transfer between neurons and glial cells, acting as a compensatory mechanism aimed at increasing the survival of neurons. Additionally, *PARKIN* knockout mice showed impaired microtubule stability and death of dopamine neurons, which also emphasizes the role of PARKIN in the regulation of mitochondrial motility [82].

In addition to regulating mitochondrial fusion, protein Mfn2 also mediates mitochondrial transport along the microtubules through interaction with Miro and Milton complexes, which promote both anterograde and retrograde mitochondrial transport. The ubiquitination of Mfn2, with the participation of the PINK1/PARKIN signaling pathway, inhibits mitochondrial trafficking [83].

The LRRK2 (Leucine-rich repeat kinase 2) gene induces the development of the dominant form of PD. The LRRK2 protein belongs to the kinase family and is located mainly in the cytosol but can also be associated with the membrane and vesicular structures of the cell, including mitochondria [84]. The study by Stafa et al. demonstrated that LRRK2 participates in the regulation of mitochondrial dynamics through interaction with dynamin family GTPases, and it phosphorylates Drp1, thereby inducing its translocation from cytosol to mitochondria. In vivo experiments conducted on brain extracts from wild-type mice have shown that LRRK2 alters the expression of the mitochondrial fission proteins Drp1, Dnm1 (Dynamin-1-like protein), and the fusion protein Opa1. In the human frontal cortex, the gene does not significantly affect the expression of Dnm1, Drp1, and Mfn2; however, it does reduce Opa1. The findings also revealed the correlation between neurite growth rate and the protein expression profile. The co-expression of LRRK2 with Mfn1 prevents excessive mitochondrial fusion and thus possible outgrowth shortening. However, Drp1-mediated shortening of neurites is not affected by LRRK2. In both instances, the attenuation of fission or the enhancement of fusion processes disrupts neuronal outgrowth [85].

Mutations in *DJ-1* (Protein deglycase DJ-1) cause the development of early-onset familial PD. Normally, the protein is localized in the cytoplasm, but under oxidative stress, it relocates to the mitochondria to provide antioxidant function. DJ-1 also ensures the maintenance of mitochondrial communication with the endoplasmic reticulum and the transfer of calcium ions between them. It was shown that a decrease in the level of DJ-1 in the cytosol causes increased fragmentation of mitochondria due to a decrease in the contact area of the organelle with the endoplasmic reticulum [12,86,87].

The most prominent area of current research interest is the investigation of alterations in mitochondrial dynamics in idiopathic forms of Parkinson’s disease. There are several reports of altered mitochondrial morphology in idiopathic PD in the cerebral cortex, substantia nigra, and caudate nucleus, where enlarged mitochondria with disrupted cristae were found [83]. Studies of mitochondrial dynamics are currently being actively conducted on experimental models of PD based on the introduction of toxins into the bodies of laboratory animals. The study by Portz et al. extends the current understanding of the relationship between the balance of mitochondrial dynamics and normal neuronal vital functions. The findings indicate that α-synucleinopathy is associated with decreased mitochondrial Drp1 function and increased mitochondrial size. Furthermore, the mitochondrial alterations observed in brain stem neurons were more pronounced than those seen in cortical neurons. [88].

The role of Drp1-mediated mitochondrial fission in the development of inflammatory processes in neurons of the olfactory bulb of rats was investigated by Zhang et al. [89]. The injection of rotenone caused significant motor impairment and decreased body weight and was accompanied by degeneration of dopaminergic neurons of the corpus striatum. The toxin has been observed to induce the active relocation of Drp1 from cytoplasm to mitochondria and their subsequent active fragmentation. The administration of a selective Drp1 inhibitor, the Mdivi-1 protein, prevented this process. To assess the inflammatory response researchers measured the levels of markers GFAP (Glial fibrillary acidic protein) and IBA1 (Ionized calcium-binding adapter molecule 1). After rotenone administration, the intensity of staining for these proteins increased; however, Mdivi-1 suppressed microglia and astrocyte activation. These findings suggest that mitochondrial dynamics are involved in pathologic changes in the dopaminergic neurons of the olfactory bulb that contribute to olfactory dysfunction in people with Parkinson’s disease.

### 1.6. Mitochondrial Dynamics as a Potential Therapeutic Target

As has already been noted, the relevance of the studies considered in this review is largely attributable to the increasing volume of data on the possibility of developing new methods of geroprotection and treatment of pathological forms of neurodegeneration through the direct modulation of mitochondrial dynamics. In the following section, a number of illustrative examples will be presented.

It is evident that numerous studies have demonstrated that positive results of certain therapeutic interventions in pathological neurodegeneration are accompanied by an adjustment of the mitochondrial dynamics balance. This was demonstrated, in particular, when the impact of physical exercise was evaluated. It is known that in AD patients these exercises have a beneficial impact on the recovery of various cognitive functions, including hippocampal memory function. To investigate the possible molecular mechanisms underlying this effect, a study was conducted on APP/PS1 transgenic mice modeling Alzheimer’s disease. The mice were subjected to interval high-intensity or continuous medium-intensity training for 12 weeks. As a result, the levels of mitochondrial fusion proteins Mfn1, Mfn2, and Opa1 were increased in the hippocampal neurons of the trained mice, while the amount of Aβ, ROS, and peroxides was reduced. Such indicators suggest a possible protective effect of physical exercise against neurodegenerative diseases and cognitive decline due to changes in mitochondrial dynamics [90].

Xu et al. demonstrated the neuroprotective effect of the long-term oral administration of spermine, spermidine, and rapamycin in SAMP8 mice modeling accelerated aging. Following a series of tests, the animals exhibited an improvement in cognitive functions after administration of these substances. At the same time, changes in mitochondrial dynamics were observed in Western blot experiments of whole-brain homogenates, showing an increase in the levels of Mfn1 and Mfn2, as well as a decrease in the ratio of phosphorylated Drp1 to the unphosphorylated form of this protein in SAMP8 mice after the consumption of spermine, spermidine, and rapamycin compared to the control SAMP8 group. Moreover, a comparison of the levels of the above markers with the data for SAMR1, which do not exhibit accelerated aging, suggests that spermidine, spermine, and rapamycin administration prevents the dysregulation of the balance between mitochondrial fission and fusion that occurs during accelerated aging [91].

The efficacy of ligustilide, the most biologically active component of the Angelica sinensis plant, in preventing neurodegenerative changes associated with Alzheimer’s disease has already been confirmed in earlier studies [92]. Zhu et al. investigated the neuroprotective effects of ligustilide using SAMP8 mice to model cognitive impairment and pathological changes in the central nervous system under accelerated aging conditions. The authors showed the restoration of the balance of mitochondrial fission and fusion in SAMP8 mice after daily administration of lingustilide for 8 weeks. Before treatment, the mice showed a shift of this balance towards fission, manifested by an increase in the level of Drp1 and a decrease in the level of Mfn1/2 in the hippocampus relative to the control group of SAMR1 mice, while after receiving lingustilide, SAMP8 mice showed increased levels of Mfn1/2 and decreased levels of Drp1. Moreover, behavioral analysis showed an improvement in memory and learning ability in such animals [93].

In addition to the positive changes in neurodegenerative processes observed in mitochondrial dynamics following specific corrective measures, a substantial body of research has been devoted to investigating the direct effects of regulators of this complex network in these conditions. One of the examples is the study of the effect of resveratrol, a well-known stimulator of mitochondrial biogenesis on neurodegeneration. In recent decades, the importance of a healthy and nutritious diet has grown considerably, and the study of the relationship between dietary intake and various diseases is becoming increasingly valuable in the elucidation of their pathogenesis. There is evidence of the effect of high-fat diets on accelerated aging and cognitive decline. The study of the effect of HF on cognitive functions was conducted on SAMP8 mice, a model of Alzheimer’s disease. Mice were divided into three groups: the control, mice on a high-fat diet, and mice on a high-fat diet treated with resveratrol. Opa1 levels in hippocampal neurons were reduced in the mice on a high-fat diet compared to both the resveratrol-treated animals and the control. Resveratrol-treated mice also performed better on cognitive tests. Thus, a high-fat diet contributes to cognitive impairment and a reduction in mitochondrial fusion proteins in the hippocampus, while resveratrol ameliorates these disorders and has strong therapeutic potential in the treatment of AD [94].

Other researchers, in an experiment on SAMP8 mice, studied the effects of the candidate drug DL0410, a derivative of phthalazinone that inhibits acetylcholinesterase and butyrylcholinesterase but differs in chemical structure from cholinesterase inhibitors such as rivastigmine, donepezil, and tacrine [95]. Mice treated with DL0410 showed improved cognitive function on the Morris water maze test, novel object recognition test, and nest building test compared to SAMP8 mice not receiving DL0410. The level of Opa1 in cortical and hippocampal neurons decreased in SAMP8 mice relative to the controls, while neurons from mice receiving DL0410 had a dose-dependent increase in this protein. These results suggest that DL0410 has therapeutic prospects in the treatment of neurodegenerative diseases, including AD [96].

The study by Gusdon et al. demonstrated an increase in the level of Drp1 in the cortex of the large hemispheres of aged 24-month-old mice after training for 17 days with daily increasing physical loads [97]. However, the levels of mitochondrial fusion markers, Mfn2 and Opa1, and PGC-1α remained unchanged. No changes in Drp1 levels were observed in a group of young 4-month-old mice exposed to the same physical activity. The authors suggest that exercise led to increased Drp1 expression and improved mitochondrial quality control and function by leveling off aging-related microRNA changes in old mice with daily exercise. In turn, the lack of changes in PGC-1α levels, a major marker of mitochondrial biogenesis, may be a consequence of exclusively aerobic neuronal metabolism, which eliminates the necessity to dramatically increase oxidative phosphorylation efficiency through increased mitochondrial mass. In support of this hypothesis, an earlier study showed an increase in PGC-1α levels in muscle tissue after exercise, which is characterized by a switch from anaerobic to aerobic metabolism [98]. On the other hand, the PGC-1α level remaining unchanged may be a consequence of a shorter duration of exercise compared to other studies where training was continued for 6–8 weeks [99,100,101].

To investigate the role of mitochondrial dynamics in the mechanism of the neuroprotective effect of leptin in Alzheimer’s disease, Cheng et al. used the HT-22 hippocampal neuron cell line. The performed immunoassays demonstrated an increase in Drp1 and Mfn1 levels after treating the cells with Aβ1-42. At the same time, combined treatment with leptin and Aβ1-42 restored the levels of Drp1 and Mfn1 to the corresponding control values. No statistically significant changes in PGC-1α level were detected in all experimental cases. The authors believe that these results indicate that PGC-1α does not play a significant role in leptin-mediated neuroprotective mechanism in AD. Furthermore, the level of Fis1 increased alongside Drp1 following the treatment of neurons with Aβ1-42, and this increase was found to normalize after leptin administration. In the authors’ opinion, this confirms the previously obtained data on the interaction of Drp1 with Fis1 and the initiation of excess mitochondrial fragmentation in AD by this complex [102].

Recent work has investigated the effects of MitoQ, an antioxidant affecting mitochondria, in in vitro and in vivo models of PD induced by 6-hydroxydopamine (6-OHDA). It was observed that 6-OHDA shifted the balance of mitochondrial dynamics toward fission by decreasing the expression of Mfn1, Mfn2, and Opa1 and increasing that of Drp1 in the dopaminergic cell line SN4741. MitoQ treatment increased Mfn2 protein and mRNA levels, promoted mitochondrial fusion, stabilized mitochondrial morphology and function, and reduced cell apoptosis. Consistent with these results, the administration of MitoQ to 6-OHDA-treated mice significantly reduced the loss of dopaminergic neurons in the substantia nigra [103]. Another study of Parkinson’s disease showed that the administration of Mdivi-1, a selective inhibitor of Drp1, significantly reduced the formation of ROS and mitophagy processes in the substantia nigra neurons of model experimental animals [104].

In this context, it is interesting to draw attention to the results indicating a direct effect on mitochondrial dynamics of some other drugs, in particular, those successfully used in endocrinology. A striking example is metformin, a widely used antidiabetic drug. Its exact mechanism of action is still poorly understood, but there is evidence that it inhibits mitochondrial fission. Several years ago, experiments on laboratory animals showed that metformin reduces the level of Drp1 with the active involvement of cellular protein kinase AMPK-a2 [105]. As a result, there was an observed reduction in mitochondrial fragmentation, the attenuation of oxidative stress, the improvement of endothelial dysfunction, the inhibition of inflammation, and the suppression of atherosclerotic manifestations in diabetes. Another example is related to pioglitazone, a drug from the thiazolidinedione family, which increases PGC1α activity and, consequently, mitochondrial biogenesis [15,106]. Moreover, as a drug, pioglitazone effectively targets the regulators of mitochondrial dynamics, Opa1, Mfn1/2, and Drp1, and has a protective effect, particularly during ischemia and inflammation, on maintaining the balance between mitochondrial fusion and fission [107,108,109]. Also, stimulatory effects on mitochondrial biogenesis have been found in other drugs such as Spirulina platensis and alogliptin (dipeptidyl peptidase-4 inhibitor) [8,110,111].

## 2. Conclusions

It is evident that disturbances in mitochondrial dynamics in nervous tissue are an almost obligatory component of both normal and pathological aging. The most significant alterations associated with age are the insufficient Mff-mediated complete division of mitochondria, leading to impaired axonal transport of the latter and, as a consequence, impaired energy supply and other mitochondria-related processes in synapses. Furthermore, it is important to highlight that changes in mitochondrial fusion processes increase with age. It can be reasonably concluded that this has a significant impact on neurogenesis, leading to a decrease in the potential of neurogenic niches and related cognitive disorders. Also, for differentiated neurons, mitochondrial fusion disorders have a pronounced negative effect, activating the processes of neurodegeneration.

Disturbances in the balance of mitochondrial dynamics can obviously play a significant pathogenetic role in neurodegenerative diseases, such as Alzheimer’s and Parkinson’s disease. The data indicating this, as considered in our review, testify to the ambiguity of the processes occurring in this case. In this context, in Alzheimer’s disease both accumulation of beta-amyloid and expression of a pathologic form of tau protein result in a reduction in the level of fusion proteins and an increase in the level of fission proteins. Concurrently, disturbances of mitochondrial dynamics may be an early indicator of this disease, suggesting a high probability of a beneficial outcome from means capable of restoring the balance of these dynamics, in particular, shifting it towards more active fusion, as well as activating mitochondrial biogenesis while still in the preamyloid phase of Alzheimer’s disease. These data, as well as similar results obtained in the study of Parkinson’s disease pathogenesis, largely explain the active growth of interest in the study of geroprotective potential of drugs that directly affect mitochondrial dynamics. In our review, we have given several examples proving the probable effectiveness of research development in this direction, but the rapid growth in the number and diversity of relevant works require a special and more detailed review analysis.

## Data Availability

All the data are shown in the main manuscript.
